# Ferroptosis and Its Potential Role in the Physiopathology of Skeletal Muscle Atrophy

**DOI:** 10.3390/ijms252212463

**Published:** 2024-11-20

**Authors:** Chen-Chen Sun, Jiang-Ling Xiao, Chen Sun, Chang-Fa Tang

**Affiliations:** 1 School of Physical Education, Hunan First Normal University, Changsha 410205, China; sunchenchen1022@hunnu.edu.cn; 2 College of Physical Education, Hunan Normal University, Changsha, 410012, China; xiaojiangling@hunnu.edu.cn (J.-L.X.); sunchen@hunnu.edu.cn (C.S.)

**Keywords:** ferroptosis, skeletal muscle atrophy, iron metabolism, lipid peroxidation, Xc-GSH-GPX4 pathway

## Abstract

Skeletal muscle atrophy is a major health concern, severely affecting the patient’s mobility and life quality. In the pathological process of skeletal muscle atrophy, with the progressive decline in muscle quality, strength, and function, the incidence of falling, fracture, and death is greatly increased. Unfortunately, there are no effective treatments for this devastating disease. Thus, it is imperative to investigate the exact pathological molecular mechanisms underlying the development of skeletal muscle atrophy and to identify new therapeutic targets. Decreased muscle mass, strength, and muscle fiber cross-sectional area are typical pathological features and manifestations of skeletal muscle atrophy. Ferroptosis, an emerging type of programmed cell death, is characterized by iron-dependent oxidative damage, lipid peroxidation, and reactive oxygen species accumulation. Notably, the understanding of its role in skeletal muscle atrophy is emerging. Ferroptosis has been found to play an important role in the intricate interplay between the pathological mechanisms of skeletal muscle atrophy and its progression caused by multiple factors. This provides new opportunities and challenges in the treatment of skeletal muscle atrophy. Therefore, we systematically elucidated the ferroptosis mechanism and its progress in skeletal muscle atrophy, aiming to provide a comprehensive insight into the intricate relationship between ferroptosis and skeletal muscle atrophy from the perspectives of iron metabolism and lipid peroxidation and to provide new insights for targeting the pathways related to ferroptosis and the treatment of skeletal muscle atrophy.

## 1. Introduction

Skeletal muscle accounts for about 40–50% of weight in humans and plays an essential role in postural support, motor control, thermogenesis, thermoregulation, and metabolism [1]. However, when stimulated by a variety of unfavorable pathological and physiological factors, the homeostasis between protein synthesis and degradation is disrupted, contributing to a rate of protein degradation that exceeds the synthesis, resulting in a decrease in muscle mass, strength, and function. This process is commonly referred to as skeletal muscle atrophy [2]. As a common muscle disease, it is characterized by a reduction in muscle mass and muscle fiber cross-sectional area (CSA) at a histological level and atrophy, which is caused by several factors including genetic mutations [Duchenne muscular dystrophy (DMD), limb-girdle muscular dystrophy (LGMD), spinal muscular atrophy (sma), amyotrophic lateral sclerosis (ALS)], aging, fasting, immobilization, malnutrition, cancer, wasting, obesity, diabetes, and chronic kidney disease (CKD) [3,4,5,6]. It seriously affects patients’ mobility and life quality [4]. Given these facts, there is an urgent need for a comprehensive and in-depth study of the pathogenesis and treatment of skeletal muscle atrophy.

**Figure 1:**
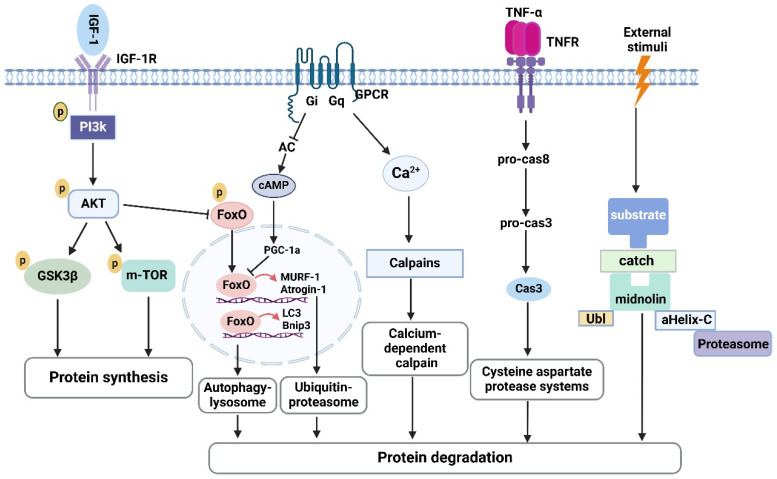
Regulatory pathways of protein synthesis and degradation. IGF/PI3K/AKT regulates protein homeostasis. IGF/PI3K/AKT/GSK3β and IGF/PI3K/AKT/m-TOR promote protein synthesis. IGF/PI3K/AKT/FOXO suppresses protein degradation. The calcium-dependent calpain system, cysteine–aspartate protease system, mandolin–proteasome pathway, ubiquitin–proteasome system (UPS), and the autophagy–lysosome pathway (ALP) promoting protein degradation.

The balance between protein synthesis and degradation determines skeletal muscle mass and function. In a healthy state, they are in dynamic balance and work together to maintain skeletal muscle mass. But when the balance is disturbed, especially when degradation is increased and/or synthesis is decreased, this will result in proteins catabolized and promote skeletal muscle atrophy [7]. As a key growth factor, insulin-like growth factor 1 (IGF-1) regulates protein synthesis and catabolism [8]. IGF mediates the phosphoinositide-3-kinase/protein kinase B (PI3K/AKT) pathway to activate glycogen synthase kinase3beta (GSK3β) and mTOR to promote muscle protein synthesis. In addition, the IGF/PI3K/AKT pathway inhibits the expression of downstream targets forkhead box O (FOXO) and atrophic genes (Murf and Atrogin-1), suppressing protein degradation and muscle atrophy [9]. Five pathways mediate protein degradation in cells: the calcium-dependent calpain system, the cysteine–aspartate protease system, the midnolin–proteasome pathway (a new ubiquitination-independent degradation pathway) [10], the ubiquitin–proteasome system (UPS) and the autophagy–lysosome system (ALP). UPS and ALP are the two main protein hydrolyzing systems involved in muscle atrophy [11]. UPS, an efficient protein degradation pathway, is responsible for the degradation of most cellular proteins. Autophagy is a form of programmed cell death, the basal form of which is necessary to maintain muscle mass and prevent atrophy, but the excessive form exacerbates muscle atrophy and causes muscle weakness [12,13] (Figure 1).

**Figure 2:**
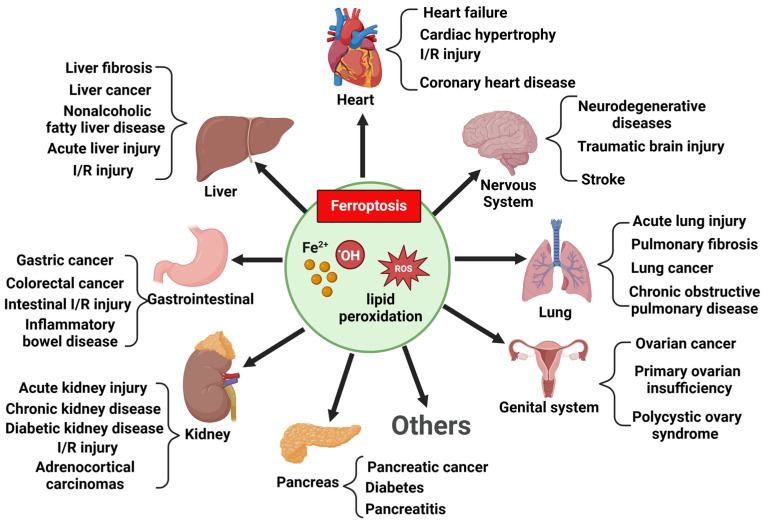
Ferroptosis has been implicated in various systemic diseases.

Several studies have attributed the imbalance between protein synthesis and degradation to increased apoptosis, oxidative stress (OS), inflammation, impaired autophagy, and mitochondrial dysfunction [14,15,16]. Recent studies have identified ferroptosis as a potentially critical factor in the initiation of many diseases, which plays an important regulatory role in pathological processes and is an essential therapeutic and prognostic target for the treatment of disease (Figure 2) [17]. It is a type of programmed cell death (PCD) characterized by lipid peroxidation accumulation and iron dependence and is morphologically and mechanistically distinct from known cell death pathways. Notably, studies in the last decade have identified key features of ferroptosis in skeletal muscle atrophy, including iron overload, decreased glutathione peroxidase 4 (GPX4) activity, the depletion of NADPH concentration, reduced levels of xCT and glutathione (GSH), and elevated lipid peroxidation [18]. This strongly suggests that ferroptosis is one of the pathological mechanisms of skeletal muscle atrophy, and targeting ferroptosis may be a promising therapeutic strategy for skeletal muscle atrophy. In this review, we discuss the mechanism of ferroptosis and sort out the progress and limitations of current studies on ferroptosis in skeletal muscle atrophy, which may provide new ideas for the treatment of ferroptosis.

## 2. Ferroptosis: A New Way of Cell Death

Cell death can be classified as accidental cell death (ACD) and PCD depending on how quickly it occurs and whether drugs or genes affect it [19]. ACD is caused by biological processes that cannot be controlled. PCD can be executed through many different subroutines, including apoptosis, autophagy, pyroptosis, necroptosis, and ferroptosis [19]. Each subroutine has different morphological features, biochemical characteristics, and functions, and has a unique molecular mechanism (Table 1) [20,21]. Ferroptosis is a unique non-apoptotic regulated cell death (RCD), and the concept was first proposed by Dixon in 2012. It is iron-dependent and caused by redox imbalance, which ultimately leads to excessive lipid reactive oxygen species (ROS) [22].

## 3. Mechanisms of Ferroptosis

**Figure 3:**
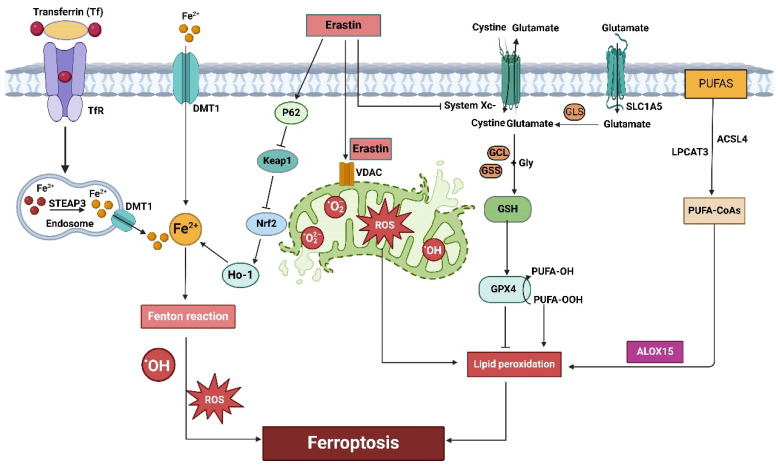
Signaling pathways that contribute to ferroptosis. (1) Transferrin (TF) binds to the transferrin receptor (TfR) to mediate Fe^3+^ entry into cells. The Fe^3+^ is reduced to Fe^2+^ by the ferriductase STEAP3, and the free Fe^2+^ is transported out of the endosome via the divalent metal transporter 1 (DMT1). Increased free Fe^2+^ in cells promotes the production of hydroxyl radicals (·OH) and ROS via the Fenton reaction, triggering ferroptosis. (2) Activating the system Xc-GSH-GPX4 pathway inhibits ferroptosis. (3) ACSL4 and LPCAT3 facilitate the conversion of PUFA to PUFA-CoAs, increasing cellular sensitivity to ferroptosis. (4) LOX (such as ALOX15) drives ferroptosis by promoting the peroxidation of PUFA. (5) Erastin inhibits the system Xc-GSH-GPX4, leading to the accumulation of lipid peroxidation and triggering ferroptosis. (6) Erastin inhibits the Keap/Nrf2/Ho-1 pathway, resulting in increased free Fe^2+^. (7) Erastin directly interacts with VDAC2/3 and induces mitochondrial dysfunction, triggering ferroptosis.

The mechanisms that regulate ferroptosis are intricate and involve numerous signaling molecules and pathways. It can be categorized into extrinsic or transporter protein-dependent pathways and the intrinsic or enzyme-regulated pathway [23]. This review briefly summarizes the major signaling pathways and key regulators of ferroptosis (Figure 3) [24].

### 3.1. The System Xc-GSH-GPX4 Pathway and Ferroptosis

The cystine/glutamate antiporter system Xc- is composed of two subunits: solute carrier family 7a member 11 (SLC7A11) and solute carrier family 3 member 2 (SLC3A2). These subunits are extensively distributed within the phospholipid bilayer. System Xc- mediates cystine into the cell for reduction to cysteine and then participates in the synthesis of glutathione (GSH). GSH, an important antioxidant, reduces intracellular ROS levels in the presence of glutathione peroxidases (GPXs). Therefore, the inhibition of system Xc- activity affects GSH synthesis, resulting in reduced GPX activity and cellular antioxidant capacity, lipid ROS accumulation, and ultimately oxidative damage and ferroptosis. GPX4, a member of the GPX family, plays a key role in inhibiting ferroptosis. It converts GSH to oxidized glutathione and reduces cytotoxic lipid peroxides (L-OOH) to the corresponding alcohols (L-OH) by using GSH as a reducing cofactor, protecting cells from ferroptosis. The inhibition of GPX4 activity or downregulation of GPX4 expression leads to the accumulation of intracellular lipid peroxides and induces ferroptosis [25,26]. The system Xc-GSH-GPX4 pathway is involved in ferroptosis by regulating amino acid metabolism, and the inhibition of this signaling pathway has been linked to the onset of various diseases, such as cancer [27] and Parkinson’s disease [28]. Therefore, the regulation of the Xc-GSH-GPX4 pathway system represents a viable strategy for the inhibition of ferroptosis.

Research has indicated that the ferroptosis inducers RAS-selective lethal 3 (RSL3) and ML162 can trigger ferroptosis by directly inhibiting GPX4 activity. This inhibition results in a diminished cellular antioxidant capacity and an accumulation of ROS [29,30]. However, interestingly, a recent study by Dorian et al. revealed that RSL3 and ML162 cannot completely inhibit the enzymatic activity of GPX4, but they were effective inhibitors of another selenoprotein, thioredoxin reductase-1 (TXNRD1) [31]. TXNRD1 inhibitors are also known to be potent inducers of cell death, such as kinnofen [32], TRi-1, and TRi-2 [33]. Therefore, the molecular mechanisms underlying the phenomena and their potential importance concerning ferroptosis need to be further explored.

### 3.2. Iron Metabolism and Ferroptosis

As an iron-dependent way of cell death, the execution of ferroptosis requires iron involvement. Iron metabolism is mainly regulated by the liver, which maintains iron homeostasis through the production and secretion of iron homeostatic regulators, including ferritin, transferrin (TF), transferrin receptor (TfR), and ferroportin (FPN). There are two stable and interconvertible forms of iron present in the cell, Fe^2+^ and Fe^3+^. TF binds the TfR to introduce insoluble Fe^3+^ into the cell, and intracellular Fe^3+^ is released by endocytosis and endosomal-lysosomal acidification. Fe^3+^ is reduced to Fe^2+^ by ferrireductase STEAP3, and free Fe^2+^ is excreted from the endosome by divalent metal transporter 1 (DMT1) [34,35].

Physiological levels of iron contribute to cell growth, whereas increased free Fe^2+^ in cells promotes the production of hydroxyl radicals (•OH) and ROS via the Fenton reaction, which triggers ferroptosis [36]. The aberrant expression of iron homeostasis regulators also affects cellular sensitivity to ferroptosis. For example, ferritin deficiency induces ferroptosis by downregulating SLC7A11 [37]. The selective autophagic degradation of ferritin triggers ferroptosis by inducing iron overload and/or lipid peroxidation [38,39]. Therefore, measuring the concentration of ferritin in serum or plasma is a simple and effective method for diagnosing iron deficiency and overload in clinics [40]. An increasing number of studies have shown that the TfR serves as a specific marker of ferroptosis [41,42]. Interestingly, TfR plays different roles in various diseases. Satellite cell-specific deletion of TfR impairs skeletal muscle regeneration through the activation of ferroptosis [43]. Whereas TfR is highly expressed in hepatocellular cancer cells and blood vessels after trauma and induces OS and iron accumulation, causing ferroptosis, and the knockdown of TfRC ameliorates ferroptosis [44,45]. Ubiquitin ligase E3 HUWE1/MULE inhibits ferroptosis by targeting and degrading TfR in acute liver injury [46]. Furthermore, ferrostatin-1 (fer-1), a ferroptosis inhibitor, attenuates LPS-induced acute lung injury by inhibiting ferroptosis [47]. These results suggest that the iron overload caused by the imbalance of iron homeostasis is the key factor in inducing ferroptosis. The inhibition of ferroptosis can be effectively achieved through the targeting of genes linked to iron overload or the application of iron chelators.

### 3.3. Lipid Metabolism and Ferroptosis

Lipid peroxidation serves as an indicator of ferroptosis. Polyunsaturated fatty acids (PUFA), such as arachidonic acid and adrenic acid, possess hydrogen atoms in the bisallylic position, which renders them susceptible to extraction by hydroxyl radicals, leading to the formation of carbon-centered radicals. The latter reacts with molecular oxygen to generate peroxyl radicals, leading to lipid peroxidation and triggering the development of cell membrane damage and ferroptosis [48]. Thus, the abundance of PUFA determines the degree of lipid peroxidation and ferroptosis. Acyl coenzyme A (CoA) synthetase long-chain family member 4 (ACSL4) and lysophosphatidylcholine acyltransferase 3 (LPCAT3) are two essential enzymes involved in the biosynthesis and remodeling of PUFAs in cell membranes. ACSL4 converts free PUFAs to PUFA-CoAs, which are subsequently re-esterified by LPCAT3 and doped into phospholipids, increasing cellular sensitivity to ferroptosis [49]. Targeting ACSL4 and LPCAT3 to reduce substrates for lipid peroxidation can increase cellular resistance to ferroptosis [48]. Lipoxygenases (LOXs) are a class of heme-free and iron-dependent dioxygenases that catalyze the dual oxidation of PUFA, leading to lipid peroxidation [50]. ALOX15, a key LOX, drives ferroptosis by promoting the peroxidation of PUFA [51]. It was found that the silencing of LOXs genes (ALOX15B or ALOXE3) prevented erastin-induced ferroptosis [50]. The downregulation of ACSL4 and 5-LOX expression inhibits ferroptosis in injured spinal cord neurons [52]. All of these experiments showed that LOXs contribute to ferroptosis. Although LOX-catalysed PUFA peroxidation increased cellular sensitivity to ferroptosis, the inhibition of LOX catalysis did not rescue the common erastin or RSL3-induced ferroptosis [53]. Thus, the contribution of LOXs to lipid peroxidation and ferroptosis remains controversial. Recent studies have found that peroxisome biogenesis genes PEX3 and PEX10 promote ferroptosis through the synthesis of polyunsaturated ether phospholipids (PUFA-ePLs) [54]. This suggests that the peroxisome may contribute to ferroptosis-related lipid peroxidation. However, more direct evidence of a correlation is needed.

In contrast to the effects of PUFAs, monounsaturated fatty acids (MUFAs) are effective in reducing the susceptibility of lipids on the plasma membrane to oxidation. Stearoyl-CoA Desaturase 1 (SCD1) and acyl-CoA synthetase long-chain family member 3 (ACSL3) are two key enzymes regulating fatty acid metabolism pathways, which mediate the generation or activation of MUFAs [55]. MUFAs alter cell membrane properties by replacing PUFAs and effectively inhibit ROS accumulation and ferroptosis [56]. It was found that exogenous MUFAs were able to reduce the sensitivity of plasma membrane lipids to lethal oxidation within a short time; however, this required the activation of MUFAs by ACSL3. In addition, exogenous MUFAs could protect cells from lipotoxicity-induced apoptosis, a process that did not require the involvement of ACSL3 [57].

### 3.4. Amino Acids Metabolism and Ferroptosis

Amino acid (AAS) metabolism is involved in the regulation of ferroptosis mainly through the regulation of the system Xc-GSH-GPX4 pathway. Other AASs involved in the regulation of lipid peroxidation, ROS, or iron metabolism were also considered to be potential ferroptosis regulators. For example, glutamine (Gln) is one of the most abundant amino acids in muscle and circulation and regulates energy supply processes [58]. Gln is metabolized by glutaminase 1 (GLS1) and glutaminase 2 (GLS2). Excessive Gln catabolism leads to lipid peroxide accumulation and triggers ferroptosis [59]. Yes-associated protein 1 inhibits ferroptosis by upregulating GLS1 in vascular smooth muscle cells [60]. The knockdown of GLS2 downregulates mitochondrial ROS levels and inhibits ferroptosis [61]. Therefore, the downregulation of intracellular Gln levels in muscle cells could be an effective strategy to resist ferroptosis.

### 3.5. Mitochondrial Dysfunction and Ferroptosis

Erastin is a classical inducer of ferroptosis that directly inhibits system Xc-lowering GSH levels. Voltage-dependent anion channels (VDACs), another target of erastin, are located in the outer mitochondrial membrane and control the transmembrane exchange of metabolites [62]. It was found that erastin directly interacts with VDAC2/3 to induce ferroptosis. This interaction results in modifications to the permeability of the outer mitochondrial membrane and a subsequent decrease in the rate of NADH oxidation [63]. Nedd4 ubiquitinates VDAC2/3 in a site-specific manner and thus inhibits erastin-induced ferroptosis in melanoma [64]. VBIT-12 protects mitochondria through the inhibition of VDAC1 oligomerization and mitigates acetaminophen-induced ferroptosis in acute liver injury [65]. VDAC has two states: open and closed. Under the open state, VDAC is primarily permeable to respiratory substrates, adenosine diphosphate (ADP), phosphoric acid (Pi), and adenosine triphosphate (ATP), allowing them to enter the mitochondria, while the closed state seals off the mitochondria [66]. The dynamic “open-closed” condition of the VDAC has a major impact on mitochondrial metabolism and function.

Iron is the most prevalent metal in mitochondria and is actively involved in maintaining normal mitochondrial functions [67]. Growing research suggests that mitochondria are important regulators of ferroptosis, and the change in its structure and function is the key feature of ferroptosis. For example, Mfrn1/2 dysregulation leads to mitochondrial iron accumulation and oxidative damage [68]. *Gpx4* deficiency leads to mitochondrial swelling, cristae reduction, and outer membrane rupture [69,70]. PPIs inhibit hepatocellular cancer progression by inducing ferroptosis through inhibition of the Nrf2/HO-1/GPX4 axis, leading to the severe impairment of mitochondrial structure and function, including a reduction in mitochondrial volume, an increase in double-membrane density, cristae deletion, and a decrease in mitochondrial membrane potential [71]. Trastuzumab causes mitochondrial dysfunction by inactivating the ErbB2/PI3K/AKT/Nrf2 pathway, increasing 4-hydroxynonenal (4-HNE) expression and VDAC1 oligomerization [72].

Mitochondria, as the primary organelles for ATP production, are essential for the control of cell survival and death. In conditions of ATP deficiency, the energy sensor AMP-activated protein kinase (AMPK) is activated. This activation influences the activity of acetyl-CoA carboxylase, which serves as the rate-limiting enzyme in fatty acid synthesis and concurrently inhibits the process of ferroptosis. This activation influences the activity of acetyl-CoA carboxylase, which serves as the rate-limiting enzyme in fatty acid synthesis and concurrently inhibits the process of ferroptosis [73,74]. Mitochondria are the major source of cellular ROS, which can induce ferroptosis by promoting lipid peroxidation. For example, arsenic induces pancreatic dysfunction and ferroptosis via the mitochondrial ROS–autophagy–lysosomal pathway [75]. Chemotherapeutic drugs induce ferroptosis via the generation of excessive ROS, which results in lipid peroxidation and mitochondrial dysfunction, ultimately contributing to ovarian damage [76]. Erastin significantly induces cell death through activation of the ROS–mitochondrial-fission–mitochondrial-autophagy pathway [77]. The inhibition of mitochondrial ROS and ferroptosis alleviates doxorubicin-induced cardiotoxicity [78]. Resveratrol mitigates ROS-induced ferroptosis through the activation of SIRT3 and compensating the GSH/GPX4 pathway [79]. Nrf2 is a key transcriptional regulator and regulates almost all genes involved in ferroptosis, including the genes for glutathione regulation, NADPH regeneration, and iron regulation. It has been found that upregulating Nrf2 to inhibit ferroptosis can delay the progress of diabetic nephropathy [80]. The activation of the Nrf2/HO-1 pathway mitigates the progression of Alzheimer’s disease by suppressing ferroptosis and neuroinflammation [81,82]. Irisin prevents sepsis-associated encephalopathy [83] and cognitive dysfunction in diabetic encephalopathy by inhibiting ferroptosis through the activation of the Nrf2/GPX4 signaling axis [84]. Interestingly, in cancer cells, NRF2 activation enhances cancer cell resistance to ferroptosis [85,86]. The overexpression of Nrf2 increases the sensitivity of glioma cells to the ferroptosis inducers erastin and RSL3 [87].

## 4. Ferroptosis and Skeletal Muscle Atrophy

Ferroptosis is a common type of cell death in models of skeletal muscle atrophy. The key feature of ferroptosis has been found in skeletal muscle atrophy caused by aging, sepsis, cisplatin, chronic kidney disease (CKD), and ALS (Table 2). Studies have confirmed that ferroptosis is a potential therapeutic target for skeletal muscle atrophy and that the inhibition of ferroptosis is effective in ameliorating and/or attenuating skeletal muscle atrophy. These results provide promising therapeutic strategies for skeletal muscle atrophy.

### 4.1. Ferroptosis and Sarcopenia

Sarcopenia is a systemic syndrome of degenerative muscle atrophy and muscle weakness characterized by age-related loss of skeletal muscle mass, strength, and function, which severely increases the risk of adverse outcomes, such as fractures, falls, impaired quality of life, and mortality in older people [103]. Malnutrition, prolonged inactivity, smoking, alcohol consumption, and chronic diseases have been shown to accelerate muscle loss. In addition to structural alterations in skeletal muscle, inflammation, mitochondrial dysfunction, oxidative stress, and other pathways are involved in the pathological process of sarcopenia [104]. Currently, the main intervention strategies proposed based on these pathophysiological mechanisms include two types: non-pharmacological and pharmacological. In non-pharmacological approaches, physical activity has been suggested as the primary treatment for sarcopenia [105]. However, there are no specific drugs approved for the treatment of sarcopenia. This suggests that the pathological mechanisms of sarcopenia need to be further explored.

As early as 2007, Altun et al. found elevated levels of iron and transferrin in the gastrocnemius muscle tissue of aging rats [106]. Two subsequent experiments have successively shown a significant increase in non-hemoglobin iron concentration (NHI) levels in the skeletal muscle of senescent rodents [107,108]. These results suggest that elevated iron load and NHI may contribute to the development of sarcopenia. However, they had no direct evidence of a relationship between ferroptosis and sarcopenia. Until recently, a study by Huang et al. demonstrated a correlation between ferroptosis and sarcopenia. They found that iron overload induced by erastin triggered ferroptosis in the skeletal muscle of sarcopenia mice and that ferric citrate was involved in iron overload-induced ferroptosis by increasing the expression of p53 and inhibiting the activity of its target gene, SLC7A11, and downregulated the expression of Gpx4, which were both reversed by Fer-1 and DFO in C2C12 cells [89]. Wang et al. found that long-term aerobic training suppressed inflammation, OS, and ferroptosis in the quadriceps muscle of aged rats through the activation of the Keap1/Nrf2 pathway [92]. However, given the diversity and complexity of factors and signals that regulate ferroptosis, more research is needed to verify the role of ferroptosis in sarcopenia.

### 4.2. Ferroptosis and CKD-Associated Muscle Atrophy

CKD is a chronic catabolic disorder characterized by progressive loss of renal mass and function, such as atrophy, fibrosis, and reduced glomerular filtration rate (GFR) [109]. According to GFR, CKD can be divided into five stages; if it is not effectively controlled during the first three stages, it will develop into end-stage renal disease. At this point, the patient will lose all renal function and must rely on renal dialysis or transplantation for survival [110]. In addition, CKD is associated with the development of many complications, including cardiovascular disease [111], skeletal muscle disease [112], anemia [113], and atherosclerosis [114]. Skeletal muscle atrophy and dysfunction are common features of CKD and are strongly associated with reduced quality of life, disability, and a high risk of death in patients [115]. Therefore, resolving muscle atrophy is critical to improving life quality and reducing adverse outcomes.

Currently, the prevailing view is that CKD-induced muscle wasting is closely related to OS, inflammation, insulin resistance, mitochondrial dysfunction, metabolic acidosis, vitamin D deficiency, intestinal flora imbalance, mucosal barrier damage, uremic toxins, and physical inactivity [110,116]. These factors together cause increased degradation and decreased synthesis of muscle proteins, leading to muscle loss, decreased muscle mass and strength, weakness, and disability. Recent studies have shown that ferroptosis plays a significant role in the development of CKD-induced muscle atrophy. Ni et al. used 5/6 nephrectomy (NPM) to construct a mouse model of CKD. They found that compared with normal mice, the levels of iron, ROS, MDA, Ptgs2, and Acsl4 were significantly increased in the skeletal muscle of NPM mice, whereas the expression of Gpx4 and GSH and GSH/GSSG ratio was reduced [91]. The results showed that oxidative damage and ferroptosis were activated in the skeletal muscle of NMP-induced CKD mice. He and Ju confirmed this [90,94]. Targeting ferroptosis and its signaling pathways may be beneficial in the treatment of CKD-related muscle damage. For example, caffeic acid inhibits ferroptosis by decreasing the levels of iron, ROS, ACSL4, and ALOX15 and increasing the levels of xCT, and GPX4 in the skeletal muscle of NPM rats [90]. Shenshuai Yingyang Jiaonang has been shown to improve muscle atrophy associated with CKD-associated muscle atrophy in rats by inhibiting ferroptosis through the HIF-1α/SLC7A11 pathway [94]. Lobetyolin inhibits ferroptosis through activation of Hedgehog-GLI1 signaling and alleviates skeletal muscle injury in 5/6 NPM mice [91]. However, it is not clear whether ferroptosis is also involved in the process of nephropathy-associated muscle atrophy induced by other factors.

### 4.3. Ferroptosis and Sepsis-Induced Muscle Atrophy

Sepsis is one of the most prevalent acute and critical conditions encountered in the intensive care unit (ICU). It is defined as an aberrant host response to infection, which leads to potentially fatal organ dysfunction [117]. Skeletal muscle atrophy is one of the most common complications of sepsis. Patients with sepsis lose 10-20% of skeletal muscle mass within a week, leading to a severe decline in physical functioning and increased mortality [118]. Therefore, elucidating the pathophysiological mechanisms of skeletal muscle atrophy is of great significance in the treatment of sepsis. Recently, Sheng et al. identified the presence of ferroptosis in the quadriceps muscle of cecum ligation- and puncture (CLP)-induced sepsis mice, as evidenced by increased expression of the ferroptosis marker proteins COX2, ACSL4, and ferritin heavy chain 1 (FTH1) and the downregulation of GPX4 expression. The suppression of STAT6 provides a protective effect against ferroptosis in CLP-induced skeletal muscle atrophy [93].

### 4.4. Ferroptosis and Cisplatin-Induced Muscle Atrophy

Cisplatin is frequently employed in the chemotherapy treatment of a range of malignancies, including but not limited to head and neck cancer, ovarian cancer, breast cancer, cervical cancer, prostate cancer, testicular cancer, bladder cancer, and lung cancer [119]. Skeletal muscle atrophy is one of the most common side effects of cisplatin chemotherapy, which is usually related to adverse reactions such as increased catabolism, weight loss, and skeletal muscle loss and is directly related to increased patient mortality [120,121]. Although low-dose cisplatin is also cytotoxic, it remains a first-line treatment for several types of tumors. Therefore, determining the pathophysiological mechanisms underlying cisplatin-induced skeletal muscle atrophy may be an important way to discover promising new therapeutic targets. A recent study by Liu et al. found that cisplatin induces inflammation, OS, and autophagy in mouse skeletal muscle and also leads to the increased expression of the ferroptosis-related genes ACSL4, HO-1, and SLC39A14. Interestingly, these pathological mechanisms are significantly exacerbated in the absence of programmed cell death protein 1 (PD-1) [95]. The results suggest that ferroptosis is one of the pathological mechanisms underlying cisplatin-induced skeletal muscle atrophy and PD-1 attenuates cisplatin-induced skeletal muscle atrophy by regulating inflammation, OS, autophagy, and ferroptosis.

### 4.5. Ferroptosis and Nerve Damage-Induced Muscle Atrophy

Wang et al. reported that GPX4 expression is decreased in ALS patients and various ALS mouse models [98], and FTH1 and NRF2 expression is downregulated in the spinal cord and forebrain of SOD1^G93A^ mice [98]. Another study also confirmed the presence of iron accumulation in the spinal motor neurons of patients with sporadic ALS [122]. The conditional ablation of *Gpx4* leads to neuronal degeneration in mice characterized by ferroptosis, increased lipid peroxidation, and mitochondrial dysfunction [88]. Yang et al. observed that SOD1^G93A^ mice exhibited increased plasma MDA concentrations and a reduction in the expression of GSH, SLC7A11, and GPX4 within both the spinal cord and the motor cortex [97]. In addition, recent studies have found iron deposition appeared after sciatic nerve injury [123]. The correlation between ferroptosis and nerve-damaging muscular atrophy was further confirmed. Thus, ferroptosis may be an important pathological mechanism in nerve-damaging muscle atrophy. Targeting ferroptosis and its associated pathways obtained promising results. For example, using the inhibitors Fer-1 and VAR10303 improved motor neurons and prolonged the lifespan of SOD1G^93A^ mice [100,101]. The overexpression of GPX4 ameliorates motor function and delays disease onset in SOD1G^93A^ mice [102]. These findings support the importance of ferroptosis in muscle atrophy associated with nerve damage. However, most of the current reports are based on the SOD1G^93A^-induced mouse model of ALS, indicating a need for further investigations to evaluate the potential therapeutic benefits.

## 5. Conclusions and Perspectives

The relationship between ferroptosis and skeletal muscle atrophy is widely recognized. The redox imbalance, disturbed iron metabolism, and accumulation of lipid peroxides involved in ferroptosis are key events that induce and initiate skeletal muscle atrophy. Currently, the presence of ferroptosis has been found in skeletal muscle induced by aging, cancer, CKD, cisplatin, sepsis, and ALS, further confirming the correlation. Furthermore, several studies have indicated that the inhibition of ferroptosis and its associated pathways can be beneficial in mitigating skeletal muscle atrophy. However, to date, research exploring the connection between ferroptosis and skeletal muscle atrophy remains in the early stages. These studies have certain limitations, such as the following. (1) The majority of research has primarily identified the presence of ferroptosis markers in atrophic skeletal muscle, while the specific role of ferroptosis in the context of skeletal muscle atrophy remains largely unexplored. (2) Although certain ferroptosis-related factors are altered in skeletal muscle atrophy, there is a lack of direct evidence regarding their role in the regulation of protein homeostasis within skeletal muscle. (3) There are many factors for skeletal muscle atrophy, and it is unclear whether other types of skeletal muscle atrophy are also involved in ferroptosis. (4) There is a lack of clinical research showing that inhibition of ferroptosis helps treat skeletal muscle atrophy patients. Thus, addressing these limitations will enhance the understanding of the role of ferroptosis in skeletal muscle atrophy and contribute to the development of innovative compounds targeting ferroptosis for the treatment of this condition.

## Figures and Tables

**Table 1:** The features and differences of ferroptosis, apoptosis, autophagy, necroptosis, and pyroptosis.

	Morphologic Features	Biochemical Features	Regulatory Signal	Key Genes	Inhibitor	Inducer
Ferroptosis	Small mitochondria with increased membrane density, outer membrane rupture, and cristae reduction in or vanishing	Iron accumulation, lipid peroxidation, and ROS excessive accumulation	Xc-/GPX4, E-cadherin-NF2-Hippo-YAP, AMPK and Hypoxia signaling, P53-SAT1-ALOX15, P62-Keap1-NRF2 pathway, FSP1-COQ10-NAD(P)H pathway, HSPB1-TRF1	GPX4, ACSL4, NRF2, TfR1, FTH1, LPCAT3, SLC7A11, SLC39A14, NCOA4, FSP1, COX2, ACSL4, P53, HSPB1	Fer-1, Liproxststatin-1, mesylate, SRS16-86, SRS11-9, Vitamin E, deferoxamine, 2,2′-pyridine Deferoxamine	Erastin, RSL3, ML162, FIN56 FINO2, Sorafenib, Sulfasalazine, (1S,3R)-RSL3, DPI7, DPI10,
Apoptosis	Cell shrinkage, membrane blebbing, nuclear fragmentation, chromatin condensation and margination, formation of apoptotic bodies, and disintegration of the cytoskeleton	DNA fragmentation	Growth factor, Nutrient deprivation, DNA damage, P53, Bcl-2, mitochondrion pathway and endoplasmic reticulum pathway, Fas ligand, TNF or TNF-related apoptosis-inducing ligand, Caspase	Cytochrome c, pro-caspase-9, pro-caspase-3, pro-caspase-7, BCL-2, BAX, BCL-X, APAF1	Nerve growth factor, fibroblast growth factor 10, metformin, resveratrol, forsythiaside B, rehmannioside A, baicalein, anthocyanins, apsinini, apigenin, delphinidin, rosmarinic acid, IGF-1	TGF-β, IL-10, IL-2, Dexamethasone, PKC-delta, Resveratrol, Curcumin, Yessotoxin, TNF family, Metal cadmium, HIF-1α
Autophagy	formation of double-membrane enclosed vesicles	Formation of double-membraned autolysosomes, including microautophagy chaperone-mediated autophagy, and macroautophagy	mTOR, AMPK, Beclin-1, P53 signaling,	Atg5/Atg7, LC3, Atg6/Beclin-1, p62/SQSTM1, Ulk-1	Rubicon, chloroquine, VPS34 inhibitors, ULK1 inhibitors, Atg4B inhibitors, Lys05, quinacrine, VATG-027, VATG-032, hydroxychloroquine	Resveratrol, Spermidine, SMER28, Luteolin, Apigenin, Salidroside, ABT-737, GX15-070 (Obatoclax mesylate), Metformin, Rapamycin and rapalogs, Curcumin, Quercetin
Necroptosis	Cytoplasmic and organelle swelling, plasma membrane rupture, pore formation on cell membranes, moderate chromatin condensation, loss of cellular and organelle integrity	Phosphorylation of MLKL by receptor RIPK1 and RIPK3, the assembly of necrosome	RIPK1/RIPK3/MLKL pathway, Fas/FasL, Toll-like receptors, TNF-R1, ROS, RIG-I and STING, PKC-MAPK-AP-1 related signaling,	RIPK1, RIPK3, FADD, MLKL, caspase-8, caspase-10	Necrostatin-1, GSK2982772, GSK’840, GSK’843, GSK’872, dabrafenib, ponatinib, pazopanib, GSK’074	Z-DNA-binding protein (ZBP1), Doxorubicin, Convallatoxin, Apurinic/apyrimidinic endonuclease 1, Cisplatin, Acetylshikonin, TNF-α, Alcohol, Tunicamycin
Pyroptosis	Cells swelling, cell membrane-forming pore, rupture, and bubbling of plasma membranes, nuclear condensation, and DNA damage	Gasdermin cleavage, gasdermin E dependent inflammasome formation, caspase-dependent, release of IL-1β and IL-18	Canonical inflammasome pathway, non-canonical inflammasome pathway, apoptotic caspases-mediated pathway, granzyme-mediated pathway	GSDMD, caspase-1, caspase-3, caspase-4, caspase-5, caspase-11, IL-1β, IL-18, NLRP3	MCC950, P2 × 7 inhibitor, silybin, dihydroquerceti, liraglutide, caspase-1 inhibitor, rosiglitazone, IL-β receptor antagonist	Triptolide, Paclitaxel, Cisplatin, Dibutyl phthalate, Copper-bacteriochlorin nanosheet, Cucurbitacin B, Simvastatin, Nobiletin, Arsenic, Metformin

YAP: Yes-associated protein; HSPB1: Heat Shock Protein Family B Member 1; NCOA4: nuclear receptor coactivator 4; FSP1: ferroptosis suppressor protein-1; COX2: cyclooxygenase 2; APAF1:apoptotic protease-activating factor 1; HIF-1α: hypoxia-inducible factor-1alpha; SMER28: Small Molecule Enhancer 28; RIPK1: receptor-interacting protein kinase 1; RIPK 3: receptor-interacting protein kinase 3; MLKL: mixed lineage kinase domain-like; GSDMD: gasdermin D.

**Table 2:** Selected research studies from 2015 to 2024 related to ferroptosis in skeletal muscle atrophy.

Feature	Comment	References
Conditional ablation of Gpx4 in neurons of mice	Increased ferritin deposition, motor neuron degeneration rapid paralysis, and severe muscle atrophy in mice	[88]
Increased iron in the skeletal muscle of old SAMP8 mice	Increased lipid peroxidation and MDA content and Ptgs2 mRNA levels; decreased NADPH and GSH content	[89]
Elevated levels of iron, ACSL4 and ALOX15, decreased xCT and GPX4 expression	Increased ferroptosis in rats with chronic kidney disease-induced muscle atrophy	[90]
Increased ferroptosis markers and lipid peroxidation products in the skeletal muscle of 5/6 nephrectomized mice	Reduced GSH/GSSG ratio, decreased GSH content, increased MDA production	[91]
Decreased FTL, FPN, and GPX4 expression in the quadriceps femoris of old rats	Decreased levels of FTL, FPN, and GPX4 in the quadriceps muscle of old rats, which were significantly increased after lifelong aerobic training	[92]
Increased levels of ferroptosis marker (COX2, ACSL4, and FTH1) and decrease GPX4 expression	Increased ferroptosis in skeletal muscle of CLP mice, inhibition of STAT6 activity attenuates ferroptosis	[93]
Elevated levels of iron and MDA and decreased NADPH, GSH, and GPX4 expression	Increased ferroptosis in rats with chronic kidney disease-induced muscle atrophy	[94]
Increased expression levels of ferroptosis ferroptosis-related genes (ACSL4, Sat1, SLC39A14)	Ferroptosis-related genes, such as ACSL4, Sat1and SLC39A14 expression levels were significantly increased in the cisplatin-treated mice atrophic muscles	[95]
Ferroptosis-related signaling pathways were significantly enriched in sarcopenia patients with chronic obstructive pulmonary disease (COPD)	A cohort study uncovers ferroptosis as a potential common mechanism of COPD complicated by sarcopenia	[96]
Elevated levels of ROS, MDA and decreased GSH, SLC7A11, and GPX4 expression	Increased ferroptosis in skeletal muscle of ALS mice induced by the SOD1^G93A^ mutation, NRF2 activation suppresses motor neuron ferroptosis	[97,98,99]
Decreased accumulation of 4HNE and iron and lipid peroxidation in ALS mice	Fe-1, VAR10303 and overexpression of GPX4 ameliorated motor neurons and prolonged lifespan in SOD1^G93A^ mice	[100,101,102]
